# Repeated stretch–shortening cycles enhance force and work despite brief deactivation between cycles

**DOI:** 10.1242/jeb.252866

**Published:** 2026-10-09

**Authors:** Tobias Elst, Sven Weidner, André Tomalka, Daniel Hahn, Wolfgang Seiberl, Florian K. Paternoster, Tobias Siebert

**Affiliations:** ^1^ Motion and Exercise Science, University of Stuttgart, 70569 Stuttgart, Germany; ^2^ Human Movement Science, Faculty of Sports Science, Ruhr University Bochum, 44801 Bochum, Germany; ^3^ School of Human Movement and Nutrition Sciences, University of Queensland, Brisbane, QLD 4067, Australia; ^4^ Human Movement Science, University of the Bundeswehr Munich, 85579 Neubiberg, Germany; ^5^ Biomechanics in Sports, Department of Sport and Health Sciences, Technical University of Munich, 80809 Munich, Germany; ^6^ Stuttgart Center for Simulation Science, University of Stuttgart, 70569 Stuttgart, Germany

**Keywords:** Stretch–shortening cycle, History dependence, Blebbistatin, Muscle stretching, Performance enhancement, Mechanosensing

## Abstract

Stretch–shortening cycles (SSCs) enhance force and mechanical work compared with pure shortening contractions and are fundamental to locomotion. Although SSC mechanics are well studied, it remains unclear whether mechanical cycle-to-cycle carryover effects persist across repeated SSCs despite brief deactivation or inhibition of cross-bridge (XB) force production. To address this, single-skinned fibres from rat extensor digitorum longus muscles (*n*=21) underwent three consecutive SSCs at a slow velocity (1% maximal shortening velocity). Fibres were activated isometrically for 20 s before SSC1, whereas SSC2 and SSC3 were each preceded by 4 s of deactivation followed by 20 s of isometric activation. Experiments were performed under control conditions and with blebbistatin to inhibit XB force production. Repeated SSCs produced progressive performance enhancements under both conditions despite brief deactivation. From SSC1 to SSC3, peak force (*F*_peak_) increased by 14.6% and total SSC work (Work_SSC_) by 50.1% under control conditions, and by 37.5% and 67.6%, respectively, with blebbistatin. Increased short-range stiffness under control conditions suggests XB-mediated stretch resistance, whereas persistent increases during XB inhibition indicate contributions from non-XB structures, particularly titin. These findings support a sarcomeric memory effect that enhances stretch resistance and work during repeated SSCs.

## Teaser

**Summary:** Performance gains across repeated stretch–shortening cycles persist despite brief deactivation or cross-bridge inhibition, revealing a sarcomeric memory that enhances stretch resistance and mechanical work.

## INTRODUCTION

Stretch–shortening cycles (SSCs), defined as active muscle lengthening immediately followed by active shortening (Cavagna et al., 1968; Komi, 2000), are fundamental features of muscle function during both cyclic and acyclic movements. SSCs underpin everyday activities and athletic performance and are, therefore, vital for basic mobility and advanced physical abilities such as walking, running and jumping (Aeles and Vanwanseele, 2019; Bosco et al., 1987; Seiberl et al., 2015). The shortening phase of SSCs has long been known to yield greater force, work and power output compared with pure shortening contractions, referred to as SSC performance enhancement, pre-stretch potentiation or, more recently, the ‘SSC effect’ (Bosco et al., 1981; Cavagna et al., 1968; Fukutani et al., 2015; Gregor et al., 1988; Seiberl et al., 2015; Tomalka et al., 2020).

The SSC effect has been documented across multiple structural scales, from *in vivo* muscle–tendon units (Goecking et al., 2024) to isolated muscle fibres (Fukutani et al., 2017; Hessel et al., 2021). Despite this consistent observation, the mechanisms underlying the SSC effect remain incompletely understood (Goecking et al., 2024; Schenau et al., 1997; Seiberl et al., 2021). For a long time, proposed mechanisms underlying the SSC effect were mainly explained by macroscopic factors such as activation dynamics (Bobbert and Casius, 2005), reflex activity (Dietz et al., 1979) and tendon elasticity (Cavagna et al., 1968; Ettema, 1996). However, in the past decade, previously rather neglected mechanisms operating at the sarcomeric level have come into focus as contributors to the SSC effect (Seiberl et al., 2015). Although an important proportion of the SSC effect during *in vivo* muscle action can be attributed to pre-activation and muscle–tendon mechanics (Goecking et al., 2024; Holzer et al., 2024), experiments on single-skinned muscle fibres (Elst et al., 2025; Fukutani et al., 2017, 2019; Tomalka et al., 2020), in which neural and tendon-based contributions are eliminated, showed that intrinsic muscle properties such as cross-bridges (XB) and non-cross-bridge (non-XB) elements underpin the SSC effect.

Compelling evidence indicates that viscoelastic non-XB elements (Colombini et al., 2016; Monroy et al., 2011; Nishikawa, 2020) play a crucial role in muscle performance enhancement (Hahn et al., 2023; Millard et al., 2024) and may therefore contribute to the SSC effect (Tomalka et al., 2020). These findings challenge traditional models in the context of history-dependent force production and highlight the complexity of muscle performance fine-tuning. Existing evidence suggests that viscoelastic non-XB elements may complement the established force-producing mechanisms and relationships described by traditional muscle models, including XB-based Huxley (Heidlauf et al., 2016; Huxley, 1957) and phenomenological Hill-type models (Hill, 1938; Millard et al., 2024). In particular, the giant protein titin, which spans the sarcomere from the Z-line to the M-band (Bang et al., 2001), has been proposed to function as a Ca^2+^-dependent viscoelastic element for which stiffness increases with activation and stretch (Joumaa et al., 2008; Labeit et al., 1997, 2003; Linke, 2018). Furthermore, it is supposed that titin-actin binding shortens titin's free length and increases its stiffness and force during active stretch (Dutta et al., 2018; Nishikawa et al., 2020; Rode et al., 2009). This mechanism provides a structural basis for performance-enhancing muscle function and elastic energy storage during dynamic actions such as SSCs. Furthermore, titin interacts with XB cycling through thick filament mechanosensing, regulating myosin head availability and thereby modulating force production (Brunello and Fusi, 2024; Desai et al., 2026; Hessel et al., 2024a; Irving, 2017; Linari et al., 2015; Marcucci, 2023). Accordingly, a detailed understanding of force production during SSCs requires understanding the interplay between XB and non-XB components. One experimental approach to isolate their respective contributions is the use of XB inhibitors. Studies manipulating XB activity during single SSCs showed that both XB and non-XB structures contribute substantially to the enhanced force and work observed during SSC shortening compared with pure shortening contractions (Fukutani et al., 2017; Fukutani and Herzog, 2020; Tomalka et al., 2020, 2021).

Although the mechanics of single SSCs are well characterised, little is known about cycle-to-cycle carryover effects and whether relative force contributions of XB and non-XB mechanisms change during multiple consecutive SSCs, as muscle force production can vary with the number of SSCs performed (Josephson, 1985). However, findings on force and work across repeated SSCs are inconsistent. For example, peak force at the end of the stretch has been reported to increase (Altman et al., 2015; Campbell and Moss, 2002), decrease (Altman et al., 2015; Hessel et al., 2021) or remain unchanged (Campbell and Moss, 2000; Lee et al., 2001). These discrepancies probably reflect differences in experimental protocols, including species, muscle type, contraction velocity and, critically, the design of the inter-cycle interval (ICI; Fig. 1). The ICI, which occurs between consecutive SSCs, determines both the duration and activation state of the muscle between SSCs.

Using an ICI in which muscles performed fixed-end contractions (15 s) under continuous maximal activation, Elst et al. (2025) demonstrated progressive increases in peak force and mechanical work with repeated SSCs. Notably, steady-state forces at the end of the ICI, immediately before the subsequent stretch onset, remained unchanged, whereas peak force at the end of the stretches increased. These performance-enhancing, cycle-to-cycle carryover effects may result from stretch-induced structural modifications during the dynamic phase, potentially involving increased stretch resistance of XBs, contributions from non-XB structures or interactions between both systems. The findings of Elst et al. (2025) raise two critical questions. First, is continuous activation during repeated SSCs required for cycle-to-cycle carryover effects? Second, are potential carryover effects affected when XBs are inhibited?

To test whether continuous activation is necessary for cycle-to-cycle carryover effects in chemically activated fibres, we introduced a brief period of deactivation during the ICI. Previous findings showed phenomenologically that stretch-induced performance enhancement can persist despite deactivation during shortening and the ICI being under electrical stimulation (Andersen et al., 2023). Based on the results from Elst et al. (2025), which showed that performance-enhancing carryover effects persisted across consecutive SSCs under continuous activation (without deactivation during the ICI), we hypothesised that brief deactivation during the ICIs might attenuate, but not abolish, these performance-enhancing carryover effects in chemically activated fibres. In addition, previous studies showed that the SSC effect can persist despite substantial reductions in XB force induced by chemical inhibitors (Fukutani and Herzog, 2021; Tomalka et al., 2024). Moreover, passive structural elements have been shown to contribute to active force during stretch (Herzog and Leonard, 2002) and to passive force enhancement, which can persist for more than 25 s after deactivation (Herzog et al., 2003). These findings suggest that non-XB structures can contribute to SSC-related force and work increases even when XB force is reduced. Accordingly, we hypothesised that cycle-to-cycle carryover effects persist under conditions of chemically suppressed XB force.

## MATERIALS AND METHODS

A detailed description of muscle fibre handling, preparation, measurement procedures and solutions used is provided in the Supplementary Materials and Methods (Table S1). Briefly, glycerinated-skinned single-fibre segments were isolated from the extensor digitorum longus (EDL) muscles of three male Wistar rats (8–10 months old, 300–350g). All procedures were approved in accordance with German animal protection law [Tierschutzgesetz, §4 (3); permit number: T 236/24].

**Fig. 1.**
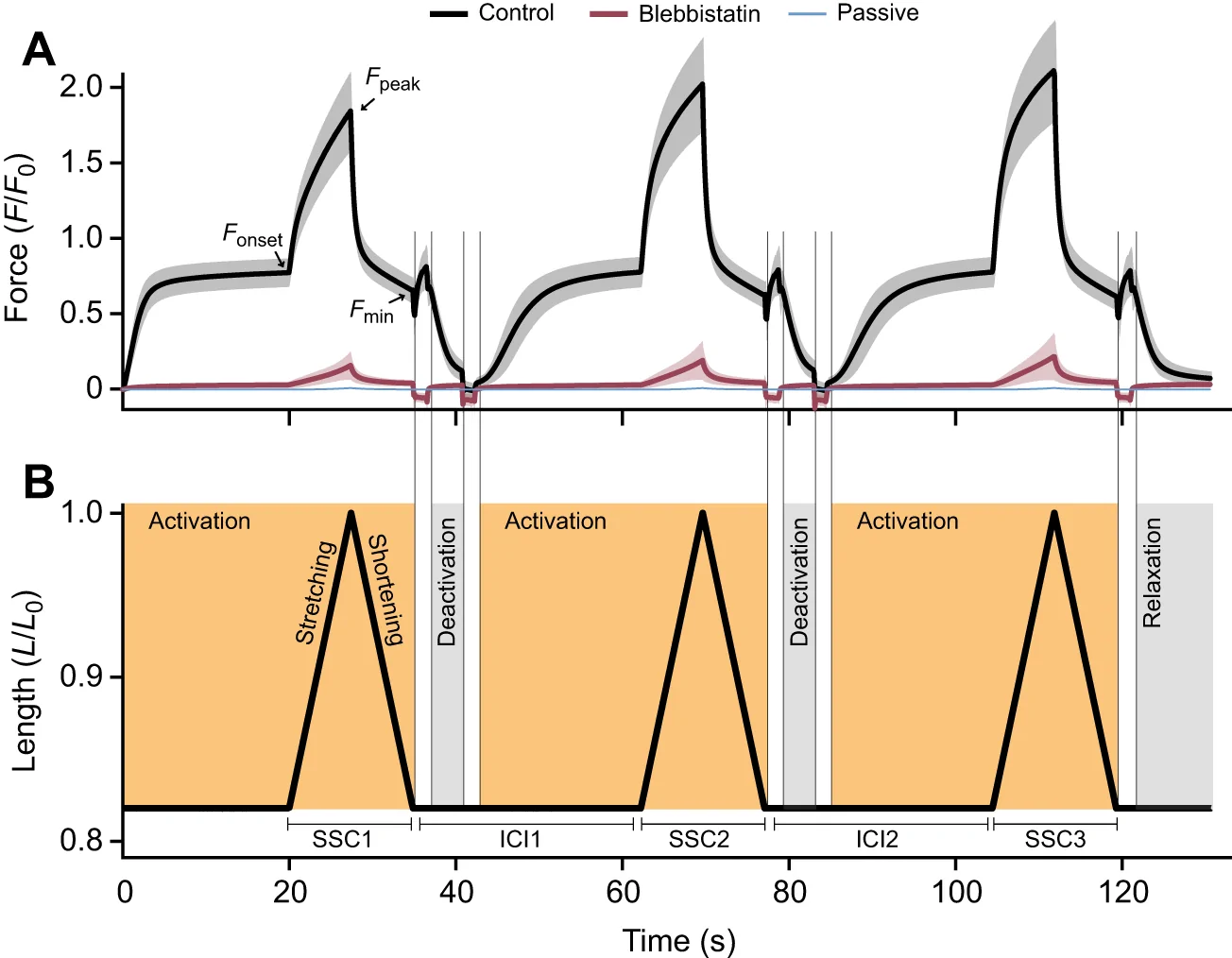
**Mean force–time and length–time traces of activated muscle fibres during repeated stretch-shortening cycles.** (A) Normalised force (*F*/*F*_0_) over time (s) of the mean response of single-skinned extensor digitorum longus (EDL) muscle fibres (*n*=21) subjected to three stretch–shortening cycles (SSCs), including the s.d. as a shaded area. Fibres were studied under control conditions (black), in the presence of blebbistatin (red) and during the passive condition (blue). For the control and blebbistatin conditions, fibres were activated by immersion in activating solution (pCa 4.5), whereas during the passive condition fibres remained continuously in relaxing solution (pCa 9.0) without bath changes. SSC experiments started with a 20 s active isometric phase, followed by three SSCs. Each SSC consisted of a stretch and a shortening phase with a stretch amplitude of 0.18 *L*_0_ and a stretch velocity of 1% *v*_max_. SSCs were separated by two identical inter-cycle intervals (ICI1 and ICI2) that consisted of briefly deactivating fibres by immersion in relaxing solution (pCa 9.0) for 4 s (grey area) and subsequently re-entering activation solution for 20 s in an isometric phase from which the next SSC starts. Steady-state force at the end of the isometric phase (*F*_onset_), peak force at the end of the stretching phase (*F*_peak_) and minimum force at the end of the shortening phase (*F*_min_) are marked and labelled for the control condition during SSC1. (B) Normalised length (*L*/*L*_0_)–time traces of the SSCs performed. Periods during which fibres were immersed in activating solution are indicated by an orange background, whereas deactivation is shown with a grey background. Bath changes (∼1 s) occurred between these intervals (white area). Intervals termed SSC1–SSC3 and ICI1–ICI2 are marked beneath the length–time trace.

### Experimental protocol

Consecutive triple SSC experiments were performed on skinned EDL fibres under two active conditions: without XB inhibition (control) and a blebbistatin-treated XB-inhibited condition. In addition to the active conditions, a passive condition was performed in relaxing solution. The purpose of the passive condition was to verify that passive forces were negligible and did not influence cycle-to-cycle effects. Each fibre first went through the passive and control conditions in randomised order, followed by irreversible treatment for the final blebbistatin condition.

The protocol covered fibre lengths from 0.82 to 1.0 optimum muscle fibre length (*L*_opt_), encompassing the ascending limb to plateau region of the force–length relationship and falling within the physiological working range of rat EDL muscle (Burkholder and Lieber, 2001). The selected fibre lengths are consistent with those used in previous studies (Elst et al., 2025; Tomalka et al., 2020, 2021, 2024; Weidner et al., 2022). The inclusion of the ascending region of the force–length relationship also provides a physiologically meaningful basis for relating the observed responses to *in vivo* SSCs of human calf muscles (Goecking et al., 2024; Holzer et al., 2024). Consecutive SSCs began with a 20 s fixed-end phase under activation at 0.82 *L*_opt_, followed by three SSCs (SSC1 to SSC3; Fig. 1). Each SSC consisted of a ramp phase in which the fibre was stretched to 1.0 *L*_opt_ and immediately shortened back to 0.82 *L*_opt_. SSCs were separated by identical ICIs (ICI1 and ICI2; Fig. 1), each consisting of 4 s of deactivation in relaxing solution (pCa 9.0; 0.82 *L*_opt_) followed by a 20 s fixed-end phase in activating solution (pCa 4.5; 0.82 *L*_opt_), which enabled the development of a steady-state force. After SSC3, fibres were relaxed for at least 420 s. Stretching and shortening were performed at a constant velocity of 1% of the maximal unloaded shortening velocity (*v*_max_). The low velocity was selected to minimise the potential confounding effects of muscle ‘give’, which has been shown to be absent under these conditions (Flitney and Hirst, 1978; Katz, 1939; Weidner et al., 2024). Consequently, this approach facilitates the isolation of carryover effects while enabling direct comparison with our previous study Elst et al. (2025). Limitations associated with the use of this low contraction velocity are discussed in the ‘Limitations’ section. The *v*_max_ of skinned EDL fibres was determined in a different experiment by Tomalka et al. (2017) on a separate set of fibres at maximum activation (*n*=5, pCa 4.5) and was found to be 2.42±0.08 *L*_opt_ s^−1^, consistent with literature values (Degens et al., 1998). Fixed-end reference contractions at 1.0 *L*_opt_ were performed to determine the maximum isometric force in the control condition (*F*_0_) and the blebbistatin condition (*F*_Bleb0_), which were used for data normalisation and to quantify the relative force suppression induced by blebbistatin. After every reference contraction, fibres were also immersed in a relaxing solution (pCa 9.0) for 420 s. To maintain structural and mechanical integrity over prolonged experiments and to minimise sarcomere inhomogeneity in maximally activated fibres, the ‘cycling protocol’ of Brenner (1983) was used during relaxation in between activations.

For the blebbistatin condition, the fibre was treated for 30 minutes with a relaxing solution containing 20 μmol l^−1^ blebbistatin (12°C, pCa 9.0). Blebbistatin was obtained from Enzo Life Sciences, Inc. (Farmingdale, NY, USA). After confirming suppression of active isometric force (*F*_Bleb0_<10% *F*_0_) in a reference contraction, the SSCs were repeated in the presence of 20 μmol l^−1^ blebbistatin in all solutions. On average, blebbistatin suppressed active forces in fixed-end reference contractions at 1.0 *L*_opt_ by 95.9±2.5% *F*_0_. This condition was, therefore, used to identify non-XB contributions to force during repeated isovelocity SSCs, consistent with previous studies (Cornachione and Rassier, 2012; Tomalka et al., 2020, 2021, 2024).

### Data processing and statistics

Force, fibre length and solution temperature were recorded at 1000 Hz using an A/D interface (604A, Aurora Scientific) and real-time software (600A, Aurora Scientific). Data analysis was performed with a custom-written MATLAB script (v.2023b; MathWorks) and MS Excel (v.1808; Microsoft). Force was expressed relative to the maximum isometric fibre force (*F*/*F*_0_), whereas fibre length was expressed relative to the optimal fibre length (*L*/*L*_opt_). Analogous to Elst et al. (2025), this study focused on three prominent force parameters (Fig. 1): the force at the end of an isometric phase immediately before stretch onset (*F*_onset_), the force at the end of the stretching phase (*F_peak_*), and the force at the end of the shortening phase (*F*_min_). *F*_onset_ was calculated as the mean force over a 0.1 s interval before stretch onset, whereas *F*_peak_ and *F*_min_ were identified as the maximum force during the stretching phase and the minimum force during the shortening phase, respectively, using MATLAB's ‘max’ and ‘min’ functions.

Mechanical work was calculated by integrating force over length using the trapezoidal method, implemented with MATLAB's ‘trapz’ function. Work was expressed as normalised values (∫*F*/*F*_0_*×*Δ*L*/*L*_0_) and defined as follows: ‘positive work’ occurs when the fibre shortens due to internally generated forces. ‘Negative work’ occurs when an active muscle is stretched by external forces (Cavagna et al., 1965). Work output was defined as work during ramp lengthening (Work_len_; Fig. 2B) and work during ramp shortening (Work_sho_; Fig. 2A). Total stretch-shortening cycle work (Work_SSC_) was then calculated by subtracting Work_sho_ from the absolute amount of work performed during stretching (Work_len_) and represents the area within the mechanical work loop during the SSC (Fig. 2A). In addition, short-range stiffness (SRS) was calculated similarly to Weidner et al. (2024) to characterise the initial resistance to stretch (Fig. 2A, zoom). SRS generally describes the disproportionately high transient initial resistance exhibited by a contracting muscle and was attributed to the elastic distortion of attached XBs, which operate as linear springs before XB detachment (Malamud et al., 1996; Morgan, 1977; Proske and Rack, 1976; Rack and Westbury, 1974). In this study, SRS was determined as the gradient value of a linear regression using MATLAB's ‘polyfit’ function over the initial 0.015 *L*_opt_ stretch, from 0.82 to 0.835 *L*_opt_, corresponding to 1.83% of the initial fibre length. This range was selected to capture the initial, steep increase in force preceding the transition to the subsequent, less steep increase observed during muscle fibre stretching (Pinniger et al., 2006).

**Fig. 2.**
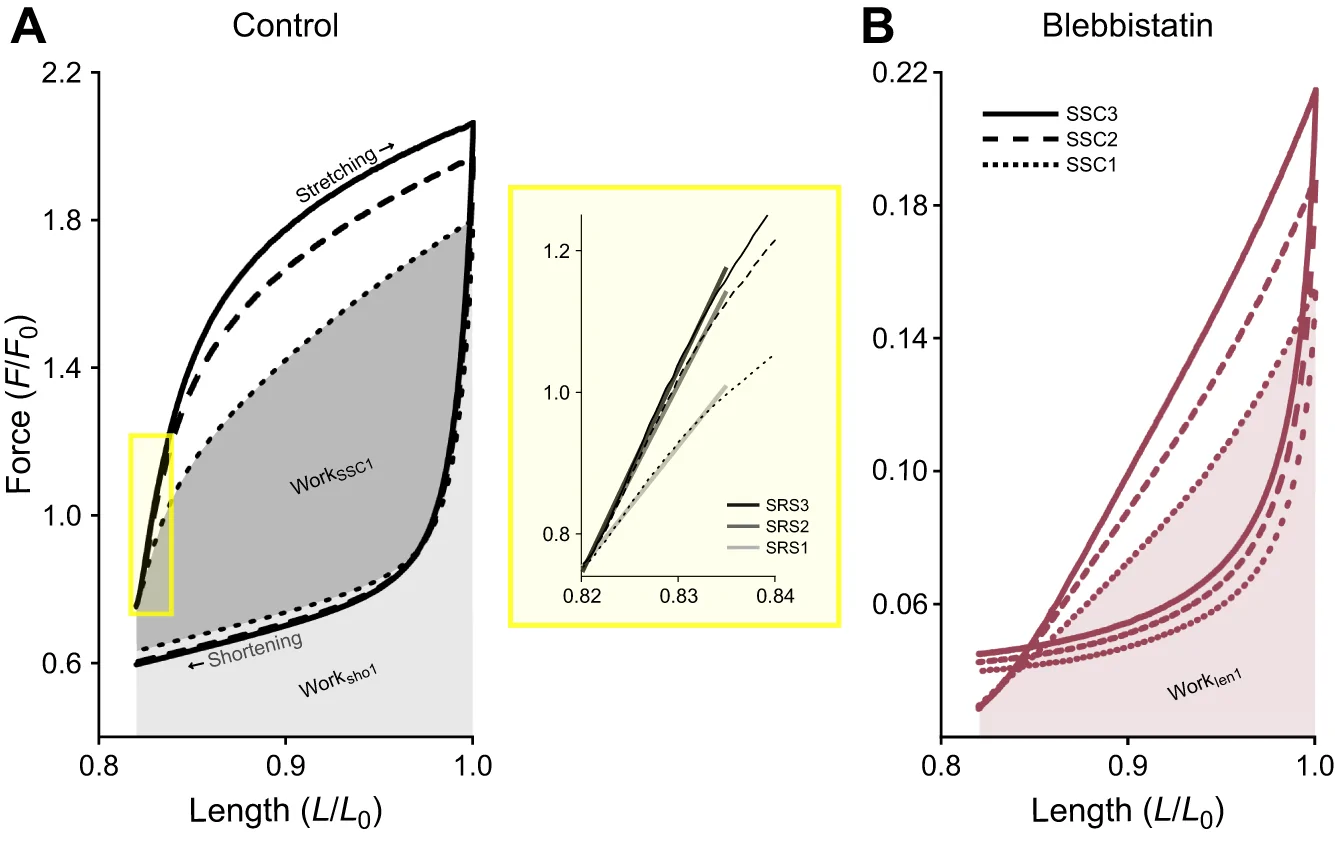
**Work loops of stretch–shortening cycles (SSCs) under different conditions.** The *y*-axis shows normalised fibre force (*F*/*F*_0_), and the *x*-axis shows normalised fibre length (*L*/*L*_0_). Each subplot contains force–length plots for three SSCs. The plots depict the mean values from 21 fibres. (A) Mean force–length traces during the control condition. The directions of stretching and shortening phases are marked by an arrow. The area used to calculate the absolute work done for the entire SSC (Work_SSC_) and the work done during the shortening phase (Work_sho_) is marked for SSC1. The inset on the right shows the linear regressions used to quantify short-range stiffness (SRS1–SRS3), fitted through SSC1–SSC3 during the initial phase of stretching. (B) Force–length traces under the blebbistatin condition, and the area used to calculate the work during the stretching phase (top traces) of the SSC (Work_len_) is shown for SSC1.

Changes in parameters between SSCs were quantified by calculating differences between mean values [e.g. *F*_peak,SSC3_–*F*_peak,SSC1_ (*F*_0_)] and relative differences between mean values [e.g. (*F*_peak,SSC3_–*F*_peak,SSC1_)/*F*_peak,SSC1_ (%*F*_0_)]. For the control and blebbistatin conditions, all mentioned parameters were considered for analysis, whereas forces in the passive condition were so small that only *F*_onset_ and *F*_peak_ were analysed.

Statistical analyses were performed on 21 tested muscle fibres. Mauchly's tests were conducted to assess sphericity. To evaluate differences within each parameter across SSC1 to SSC3, repeated measures (RM) ANOVAs were conducted. When the assumption of sphericity was violated, a Greenhouse–Geisser correction was applied. *Post hoc* analyses were performed using pairwise comparisons if the ANOVAs revealed significant differences. No correction for multiple comparisons was applied to the *post hoc* tests, and exact *P*-values were reported for interpretation of the data. Statistical analysis was performed using SPSS v.29 (IBM), with the significance level set at *P*=0.05. Figures and tables were created using MATLAB (v.2023b; MathWorks), Inkscape (v.1.3.2; Inkscape Project) and MS Excel (v.1808; Microsoft).

## RESULTS

Isometric activation of the fibres produced a gradual rise in force up to a steady state (Fig. 1, *F*_onset_). During active stretching, force increased sharply and continued to rise until the end of the stretch, resulting in a distinct peak (Fig. 1, *F*_peak_). During the subsequent shortening phase, force declined substantially and reached a local minimum by the end of shortening (Fig. 1, *F*_min_). Following this, fibres were transferred to the relaxing solution, resulting in their deactivation and a visible decay in force over the subsequent 4 s. Fibres almost fully relaxed during this period. For example, in the control condition, when the fibres re-entered the activating solution in ICI1 and ICI2, force began rising back into a steady state from 0.042±0.037 *F*_0_ and from 0.044±0.038 *F*_0_, respectively. Overall, across both control and blebbistatin conditions, peak forces and mechanical work increased over multiple SSCs, despite brief deactivation between cycles. SRS (Fig. 2A, zoom) increased with repetitive SSCs in the control condition but remained unchanged in the blebbistatin condition.

### Control condition

RM ANOVA showed no differences in *F*_onset_ between consecutive SSCs for the control condition (*n*=21) (*F*_1.18,23.57_=0.07, *P*=0.837) (Fig. 3A, Table 1). In contrast, differences in *F*_peak_ were observed across SSCs (*F*_1.04, 20.81_=64.95, *P*<0.001). *Post hoc* analysis revealed that *F*_peak_ increased from SSC1 to SSC2 (+9.5%, *P*<0.001), from SSC2 to SSC3 (+5.1%, *P*<0.001), and for the overall change from SSC1 to SSC3 (+14.6%, *P*<0.001) (Fig. 3C, Table 1). Likewise, RM ANOVA revealed differences in *F*_min_ across cycles (*F*_1.42,28.43_=20.51, *P*<0.001). Pairwise comparisons showed a significant decrease in *F*_min_ from SSC1 to SSC2 (−4.8%, *P*<0.001), whereas the subsequent change from SSC2 to SSC3 was not significant (−1.1%, *P*=0.100). Overall, *F*_min_ decreased by −5.9% from SSC1 to SSC3 (*P*<0.001) (Fig. 3E, Table 1). Differences were also observed for SRS with consecutive cycling (*F*_1.12,22.30_=72.46, *P*<0.001). *Post hoc* analysis revealed that SRS increased from SSC1 to SSC2 (+54.2%, *P*<0.001), from SSC2 to SSC3 (+13.9%, *P*<0.001), resulting in an overall increase (+68.1%, *P*<0.001) from SSC1 to SSC3 (Fig. 2A, zoom; Table 2).

**Fig. 3.**
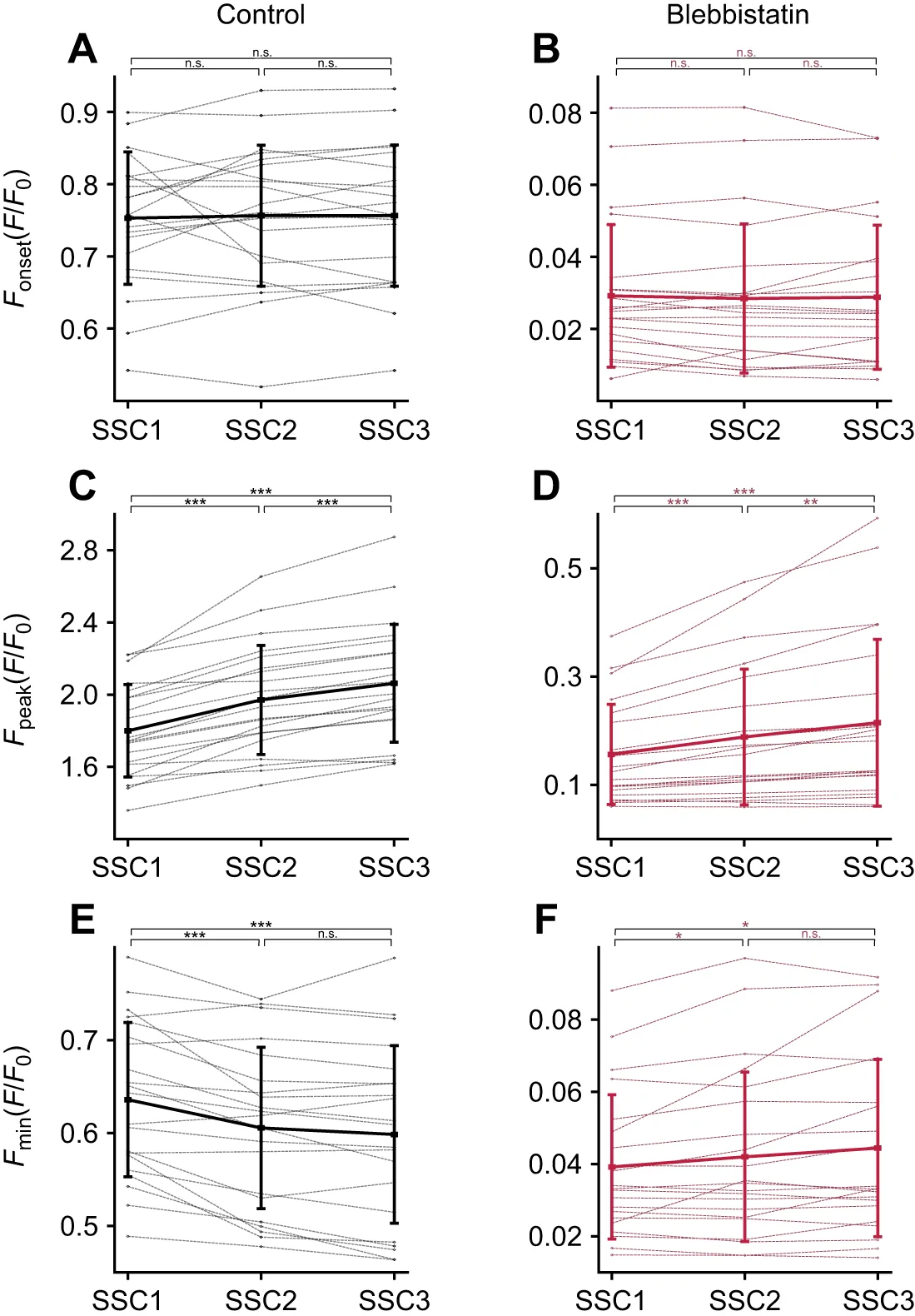
**Comparative analysis of force immediately before stretch onset (*F*_onset_), peak force at the end of the stretching phase (*F*_peak_), and minimum force at the end of the shortening phase (*F*_min_) across three consecutive SSCs.** The *y*-axis represents normalised force (*F*/*F*_0_), while the *x*-axis indicates the three consecutive cycles (SSC1, SSC2 and SSC3). Each subplot contains individual data from 21 fibres, with thin lines representing single fibres. Bold lines indicate means±s.d. Statistical significance is indicated: ***P<0.001; **P<0.01; *P<0.05; n.s., no significant differences. (A,B) *F*_onset_ for the control condition (A) and blebbistatin condition (B). No significant differences in *F*_onset_ were observed across SSCs for both conditions. (C,D) *F*_peak_ for the control condition (C) and blebbistatin condition (D). *F*_peak_ increased progressively from SSC1 to SSC3 in both conditions. (E,F) *F*_min_ for the control condition (E) and blebbistatin condition (F). *F*_min_ decreased from SSC1 to SSC3 in the control condition and increased across cycles in the blebbistatin condition.

**Table 1.** Descriptive statistics and pairwise comparison of onset force (*F*_onset_), peak force (*F*_peak_), minimum force (*F*_min_), absolute work of the entire SSC (Work_SSC_), shortening work (Work_sho_) and stretching work (Work_len_) per cycle

	Descriptive statistics				Difference in mean values				
	Condition	Cycle	Mean	s.d.	Comparison	Total	Relative %	95% CI	*P*-value
Onset force (*F*_onset_/*F*_0_)	Control	SSC1	0.753	0.092	SSC1/SSC2	0.003	0.4	–	–
		SSC2	0.756	0.098	SSC2/SSC3	0.000	0.0	–	–
		SSC3	0.757	0.098	SSC1/SSC3	0.004	0.5	–	–
	Blebbistatin	SSC1	0.029	0.020	SSC1/SSC2	−0.001	−2.5	–	–
		SSC2	0.028	0.021	SSC2/SSC3	0.000	1.3	–	–
		SSC3	0.029	0.020	SSC1/SSC3	0.000	−1.3	–	–
Peak force (*F*_peak_/*F*_0_)	Control	SSC1	1.800	0.256	SSC1/SSC2	0.171	9.5	0.125–0.217	<0.001
		SSC2	1.971	0.302	SSC2/SSC3	0.092	5.1	0.069–0.115	<0.001
		SSC3	2.063	0.326	SSC1/SSC3	0.263	14.6	0.196–0.330	<0.001
	Blebbistatin	SSC1	0.156	0.092	SSC1/SSC2	0.032	20.6	0.016–0.049	<0.001
		SSC2	0.188	0.126	SSC2/SSC3	0.026	16.9	0.011–0.042	<0.01
		SSC3	0.215	0.154	SSC1/SSC3	0.059	37.5	0.027–0.090	<0.001
Minimum force (*F*_min_/*F*_0_)	Control	SSC1	0.636	0.083	SSC1/SSC2	−0.030	−4.8	−0.044 to −0.017	<0.001
		SSC2	0.606	0.087	SSC2/SSC3	−0.007	−1.1	−0.015–0.001	0.100
		SSC3	0.599	0.096	SSC1/SSC3	−0.037	−5.9	−0.053 to −0.021	<0.001
	Blebbistatin	SSC1	0.039	0.020	SSC1/SSC2	0.003	7.1	0.000–0.005	<0.05
		SSC2	0.042	0.023	SSC2/SSC3	0.002	6.2	0.000–0.005	0.082
		SSC3	0.044	0.025	SSC1/SSC3	0.005	13.3	0.001–0.009	<0.05
Work_SSC_ (∫*F***/***F*_0_Δ*L/L*_opt_)	Control	SSC1	110.58	28.20	SSC1/SSC2	39.2	35.5	31.0–47.5	<0.001
		SSC2	149.79	34.91	SSC2/SSC3	16.2	14.6	12.3–20.1	<0.001
		SSC3	165.98	38.22	SSC1/SSC3	55.4	50.1	43.8–67.0	<0.001
	Blebbistatin	SSC1	4.65	3.86	SSC1/SSC2	1.8	38.8	0.8–2.8	<0.01
		SSC2	6.46	5.54	SSC2/SSC3	1.3	28.8	0.5–2.2	<0.01
		SSC3	7.80	6.93	SSC1/SSC3	3.1	67.6	1.3–5.0	<0.01
Work_sho_ (∫*F***/***F*_0_Δ*L/L*_opt_)	Control	SSC1	145.25	16.83	SSC1/SSC2	−2.5	−1.7	–	–
		SSC2	142.77	17.91	SSC2/SSC3	−0.1	−0.1	–	–
		SSC3	142.69	19.25	SSC1/SSC3	−2.6	−1.8	–	–
	Blebbistatin	SSC1	10.22	5.11	SSC1/SSC2	1.2	11.5	0.5–1.9	<0.01
		SSC2	11.40	6.28	SSC2/SSC3	1.0	9.3	0.2–1.7	<0.05
		SSC3	12.35	6.96	SSC1/SSC3	2.1	20.8	0.8–3.5	<0.01
Work_len_ (∫*F***/***F*_0_Δ*L/L*_opt_)	Control	SSC1	−255.83	34.01	SSC1/SSC2	−36.7	14.4	−46.4 to −27.0	<0.001
		SSC2	−292.56	41.73	SSC2/SSC3	−16.1	6.3	−20.2 to −12.0	<0.001
		SSC3	−308.67	45.13	SSC1/SSC3	−52.8	20.7	−65.8 to −39.8	<0.001
	Blebbistatin	SSC1	−14.87	8.07	SSC1/SSC2	−3.0	20.0	−4.6 to −1.4	<0.001
		SSC2	−17.85	10.71	SSC2/SSC3	−2.3	15.4	−3.7 to −0.9	<0.01
		SSC3	−20.14	12.79	SSC1/SSC3	−5.3	35.5	−8.1 to −2.4	<0.01

Note that comparisons are for *F***_0_** in onset, peak and minimum force conditions. For *F*_onset_ under the control and blebbistatin conditions and for Work_sho_ under the control condition, ANOVAs did not indicate a significant main effect. Consequently, no *post hoc* tests were performed and *P*-values are not reported (indicated by –). All ∫*F***/***F*_0_Δ*L/L*_opt_ values are shown ×10^3^.

**Table 2.** Descriptive statistics and pairwise comparison of short-range stiffness (SRS1–SRS3) corresponding to SSC1–SSC3 in control and blebbistatin conditions

	Cycle	Gradient $\left(\frac{F/F_{0}}{L/L_{0}}\right)$	*R* ^2^	Comparison	Total difference	Relative difference	*P*-value
Control	SSC1	17.2 (±5.0)	0.996	SSC1/SSC2	9.3	54.1%	<0.001
	SSC2	26.5 (±5.9)	0.997	SSC2/SSC3	2.4	13.9%	<0.001
	SSC3	28.9 (±6.4)	0.997	SSC1/SSC3	11.7	68.1%	<0.001
Blebbistatin	SSC1	0.52 (±0.62)	0.998	SSC1/SSC2	0.09	18.0%	–
	SSC2	0.61 (±0.73)	0.994	SSC2/SSC3	−0.05	−9.3%	–
	SSC3	0.56 (±0.73)	0.996	SSC1/SSC3	0.05	8.7%	–

Gradient values are means±s.d. ANOVA did not indicate a significant main effect for the blebbistatin condition. Consequently, no *post hoc* tests were performed, and *P*-values are not reported (indicated by –).

Regarding mechanical work, Work_SSC_ increased across cycles (*F*_1.05,20.92_=97.01, *P*<0.001). Consecutive cycling resulted in a 35.5% increase from SSC1 to SSC2 (*P*<0.001), a 14.6% increase from SSC2 to SSC3 (*P*<0.001) and a total increase of 50.1% from SSC1 to SSC3 (*P*<0.001) (Fig. 2A, Table 1). Work_sho_ remained statistically unchanged over the three SSCs, as indicated by the RM ANOVA (*F*_1.27,25.30_=2.40, *P*=0.128) (Fig. 2A, Table 1). Finally, an effect on Work_len_ was observed (*F*_1.06,21.22_=68.44, *P*<0.001). Work_len_ increased by 14.4% from SSC1 to SSC2 (*P*<0.001), by 6.3% from SSC2 to SSC3 (*P*<0.001) and by a total of 20.7% from SSC1 to SSC3 (*P*<0.001) (Fig. 2A, Table 1).

### Blebbistatin condition

Similarly to the control condition, RM ANOVA showed no differences in *F*_onset_ between SSCs for the blebbistatin condition (*n*=21) (*F*_2.00,40.00_=0.34, *P*=0.714) (Fig. 3B, Table 1). However, differences in *F*_peak_ were attained across SSCs (*F*_1.03,20.64_=14.99, *P*<0.001). Pairwise comparisons indicated that *F*_peak_ increased by 20.6% from SSC1 to SSC2 (*P*<0.001), by 16.9% from SSC2 to SSC3 (*P*<0.01) and by 37.5% from SSC1 to SSC3 (*P*<0.001) (Fig. 3D, Table 1). Significant differences in *F*_min_ were also observed across the three SSCs (*F*_1.37,27.39_=5.44, *P*<0.05). Subsequent *post hoc* analysis revealed an increase in *F*_min_ from SSC1 to SSC2 (+7.1%, *P*<0.05), no significant change from SSC2 to SSC3 (+6.2%, *P*=0.082) and an overall increase of 13.3% from SSC1 to SSC3 (*P*<0.05) (Fig. 3F, Table 1). SRS remained unchanged across cycles (*F*_2,40_=1.34, *P*=0.272) (Table 2).

Work_SSC_ showed a significant increase across cycles (*F*_1.03, 20.57_=13.03, *P*<0.01). Specifically, Work_SSC_ increased from SSC1 to SSC2 by 38.8% (*P*<0.01), and from SSC2 to SSC3 by 28.8% (*P*<0.01), resulting in an overall increase of 67.6% from SSC1 to SSC3 (*P*<0.01) (Fig. 2B, Table 1). The results of the RM ANOVA also showed significant differences in Work_sho_ between consecutive SSCs (*F*_1.15, 23.05_=10.49, *P*<0.01). Pairwise comparisons indicated an increase of 11.5% from SSC1 to SSC2 (*P*<0.01), an increase of 9.3% from SSC2 to SSC3 (*P*<0.05) and a total increase of 20.8% from SSC1 to SSC3 (*P*<0.01) (Fig. 2B, Table 1). Finally, a significant effect was observed for Work_len_ (*F*_1.05, 20.93_=14.51, *P*<0.001). Work_len_ increased by 20.0% from SSC1 to SSC2 (*P*<0.001) and by 15.4% from SSC2 to SSC3 (*P*<0.01), resulting in a total increase of 35.5% from SSC1 to SSC3 (*P*<0.01) (Fig. 2B, Table 1).

### Passive condition

During the passive condition (no activation; pCa 9.0), *F*_onset_ was on average 0.00007±0.00021 *F*_0_ preceding SSC1, 0.00012±0.00045 *F*_0_ preceding SSC2 and 0.00014±0.00108 *F*_0_ preceding SSC3. RM ANOVA revealed no statistical differences in *F*_onset_ with consecutive cycling (*F*_1.11,22.16_=0.11, *P*=0.773). *F*_peak_ was on average 0.00938±0.00471 *F*_0_ in SSC1, 0.00927±0.00454 *F*_0_ in SSC2 and 0.00950±0.00459 *F*_0_ in SSC3, and did not change across cycles (*F*_1.24, 24.82_=0.88, *P*=0.379). Since *F*_onset_ and *F*_peak_ are minimal and did not change with consecutive cycling, we consider them negligible and will therefore not consider them for further analysis.

## DISCUSSION

### Overview

This study investigated whether performance-enhancing cycle-to-cycle carryover effects persist during repeated SSCs despite brief deactivation phases and suppression of XB force production by blebbistatin. *F*_peak_, Work_SSC_ and Work_len_ increased from SSC1 to SSC3 under both conditions, whereas SRS increased only in the control condition. These findings suggest that cycle-to-cycle carryover effects reflect a cumulative ‘sarcomeric memory’ that can persist independently of continuous activation and intact XB cycling.

### Performance-enhancing carryover effects can survive brief deactivation

Previous studies have reported increases, decreases or no change in mechanical performance across repeated SSCs. These divergent findings probably reflect differences in activation timing, inter-contraction interval (ICI) and muscle preparation (Altman et al., 2015; Andersen et al., 2023; Campbell and Moss, 2000, 2002; Elst et al., 2025; Hessel et al., 2021; Lee et al., 2001; Morgan et al., 1996). In particular, the precise timing of activation within the SSC itself strongly influences the cycle-to-cycle force response (Sawicki et al., 2015).

Our results show that *F*_peak_ and Work_SSC_ progressively increased across repeated SSCs despite the introduction of brief deactivation periods in the ICI. These findings are consistent with previous data obtained under continuous activation from Elst et al. (2025) and Campbell and Moss (2002), both of which used long ICIs (>5 s) and similarly reported unchanged *F*_onset_ alongside progressively increasing SSC performance. Compared with Elst et al. (2025), the magnitude of the carryover effect observed in the present study was slightly smaller (+15% increase in *F*_peak_ from SSC1 to SSC3 versus 18% in Elst et al., 2025), indicating that brief deactivation attenuated, rather than abolished, the effect.

In contrast, the progressive decline in *F*_onset_ accompanied by increasing *F*_peak_ observed by Andersen et al. (2023) in whole rat EDL muscle following repeated active stretching may reflect protocol-specific differences. Alongside the aforementioned significance of the activation/deactivation timing within the ‘SSC’ itself, electrical stimulation and fatigue, as well as whole-muscle phenomena such as biaxial aponeurosis deformation and loading (Coenning et al., 2024; Ross et al., 2026), and muscle–tendon unit decoupling (Holzer et al., 2024), may alter fibre-level contraction dynamics and complicate direct comparisons across studies. Overall, continuous activation between SSCs is likely not a prerequisite for performance-enhancing cycle-to-cycle carryover effects. Instead, active muscle stretch appears to induce mechanical changes that persist through a brief period of deactivation and affect the subsequent SSC.

### Contributions of cross-bridges

SRS increased substantially across repeated SSCs under control conditions but remained unchanged under blebbistatin (Table 2). Previous studies have shown that SRS depends strongly on the duration of the ICI. SRS is reduced when ICIs are short (<5 s), but increases progressively with longer inter-stretch intervals, up to at least 10 s, where it is usually fully recovered (Campbell and Moss, 2002; Horslen et al., 2023; Rassier and Herzog, 2004). Furthermore, Campbell and Moss (2002) reported that at submaximal activation levels (pCa 6.2 and 5.8), SRS during a second SSC was greater than during the first SSC (their fig. 8).

Increased SRS at maximal activation is unlikely to arise from changes in XB stiffness or the number of strongly bound XBs, as stretch velocity was unchanged across SSCs and *F*_onset_ remained constant (Ford et al., 1977; Lombardi and Piazzesi, 1990; Morgan, 1977). Therefore, an additional explanation is a progressive increase in low-force XBs, which contributed relatively little to steady-state isometric force but may resist the subsequent stretch, thereby increasing SRS. These low-force XBs could include weakly bound XBs or strongly bound XBs in low-force mechanical states (Fitts, 2008; Månsson, 2025; Seow et al., 1997).

Thick-filament mechanosensing provides a potential mechanism for such a redistribution of XB states. Mechanical loading of titin and myosin-binding protein C during stretch may promote the recruitment of myosin heads from the OFF state towards the ON state, a configuration that favours actin interactions (Hessel et al., 2024a; Squarci et al., 2023). In addition, stretch-induced changes in lattice spacing could additionally increase the probability of actin–myosin interactions (Hessel et al., 2022). Such transient changes in lattice spacing during stretch could also trigger structural or mechanosensory changes that persist throughout the subsequent shortening phase. This could improve longer-lasting longitudinal alignment of myosin heads in the fibre direction, such that the same force generated within the myosin could produce greater SRS in the fibre direction. Reduction in lattice spacing may also allow a greater and potentially faster transition of myosin heads from the ON state into the weakly bound state. Furthermore, longer-lasting positioning of tropomyosin in an intermediate (closed) state on actin may permit more frequent and longer-lasting binding of weak XBs (Moretto et al., 2022; Rassier and Månsson, 2025). Consequently, the increase in the number of low-force XBs would not necessarily increase force but could contribute to greater SRS. This would also account for the effect being predominantly triggered by the first SSC and diminishing increments in subsequent cycles. Further investigations of XB states and forces during repeated SSCs are required to clarify this mechanism.

The increase in SRS provides a plausible explanation for the concomitant increases in *F*_peak_, Work_len_ and consequently, Work_SSC_, observed in the control condition. Consistent with this interpretation, unchanged or increasing peak forces are more commonly observed when SSCs are preceded by sufficiently long active steady-state phases (Campbell and Moss, 2002; Elst et al., 2025), allowing maximal isometric force to develop through the formation of a maximal number of XBs.

Moreover, changes in *F*_peak_ can be affected by XB viscosity (Altman et al., 2015; Hessel et al., 2021). Altman et al. (2015) showed that high-frequency cycling (10 Hz) without ICIs induced thixotropic reductions in peak force, whereas low-frequency cycling (0.25 Hz) promoted rheopectic increases. When XB force production was chemically suppressed, however, *F*_peak_ increased across all tested frequencies (0.25–10 Hz). Together with the present findings, these results suggest that both ICI duration and stretch velocity may modulate cycle-to-cycle carryover at the XB level.

### Contribution of non-cross-bridge structures

The blebbistatin condition revealed a complementary component of the carryover effect. Despite preventing strong XB force generation, *F*_peak_, Work_SSC_ and Work_len_ increased progressively across repeated SSCs. Although the absolute increase in *F*_peak_ was greater in the control condition, the larger relative increase under blebbistatin (+38% versus +15%; Table 1), together with the selective increases in *F*_min_ and Work_sho_, suggests that non-XB structures possess an intrinsic capacity to enhance SSC performance not only during stretch but also during shortening. These findings suggest that non-XB structures do not merely augment an XB-driven response but can themselves generate a substantial carryover effect, which is normally at least partially masked by active force-generating XBs.

Among the candidate non-XB structures, titin provides the most coherent mechanistic framework for the observed carryover. As a calcium-sensitive molecular spring (Dutta et al., 2018; Herzog, 2019a; Labeit et al., 2003; Leonard and Herzog, 2010; Nishikawa, 2020; Tomalka, 2023), titin increases its stiffness through calcium binding (Joumaa et al., 2008; Labeit et al., 1997) and probably also by calcium-dependent interactions with actin (Dutta et al., 2018; Linke et al., 2002; Powers et al., 2016; Yamasaki et al., 2001), which shortens its effective free spring length (Dutta et al., 2018; Nishikawa et al., 2020; Rode et al., 2009). Importantly, the persistence of these calcium- and actin-dependent interactions after fibre deactivation (Bianco et al., 2007; Joumaa et al., 2007) provides a plausible explanation for how a preceding stretch could continue to influence the mechanical response of the subsequent SSC. Other structures, including nebulin (Kawai et al., 2018), microtubules (Kerr et al., 2015) and myosin-binding protein C (Moos, 1981), also contribute to passive stiffness. However, titin is currently the strongest candidate for explaining the calcium- and actin-dependent stiffening observed here, given its known ability to exhibit both stiffening properties (Herzog, 2019b). Dynamic increases in titin stiffness therefore provide a plausible mechanistic basis for part of the progressive increase in *F*_peak_ (Rode et al., 2009) and highlight a complex interaction between XB and non-XB components across repeated cycles (Hessel et al., 2021, 2024b). The increase in Work_sho_ and *F*_min_ under blebbistatin suggests a progressive stiffening of non-XB elements that allows greater recovery of stored elastic energy during shortening once XB force production is minimised. This interpretation agrees with previous evidence that calcium-sensitive non-XB viscoelastic structures contribute substantially to SSC force and work (Hessel et al., 2021; Tomalka et al., 2020).

### The ‘sarcomeric memory’ idea

Together, these findings support an idea in which active stretching establishes a ‘sarcomeric memory’ in repeated SSCs. This memory probably comprises two interacting components: a progressively stiffening, calcium-sensitive non-XB structure, with titin being the most plausible candidate and a modest increase in the population of low-force XBs. XB-dependent force depression during shortening may partially mask the positive contribution of non-XB structures under control conditions, signalling that both XBs and non-XBs are unlikely to act independently. Rather, they appear to represent interacting elements of a single ‘sarcomeric memory’ that progressively enhances SSC performance across repeated cycles, with the effect probably saturating at some level.

### Functional and comparative significance

Persistence of stretch-induced mechanical changes across contractions could allow muscle fibres to retain part of the benefit of a preceding contraction without requiring a proportional increase in neural activation. Repeated SSCs in the present study progressively increased stretch resistance and energy absorption. Such behaviour may be functionally relevant during tasks that require muscles to repeatedly decelerate movement, absorb impact energy or resist external perturbations.

At the level of the whole muscle–tendon unit, however, this ‘sarcomeric memory’ would not operate in isolation. Rather, it would likely interact with tendon stretch and recoil, as well as the storage and return of elastic energy (Cavagna et al., 1968; Goecking et al., 2024; Holzer et al., 2024; Komi, 2000), thereby contributing alongside these mechanisms to overall SSC performance. Our findings also highlight that the muscle's intrinsic capacity to absorb energy during stretch, reflected here by Work_len_, deserves consideration. Beyond contributing to subsequent force production, this capacity may improve the ability of muscle to act as an effective mechanical brake and finely regulate force during unexpected perturbations without relying exclusively on comparatively slow sensorimotor feedback (Araz et al., 2023; Daley and Biewener, 2006).

Titin-based calcium- and stretch-dependent stiffening is found across vertebrate striated muscles and is not unique to rat EDL fibres (Linke, 2018; Nishikawa, 2020). The proposed ‘sarcomeric memory’ may therefore represent a general feature of muscle, although its magnitude and functional role are likely to vary. More broadly, these findings add to growing evidence that titin-based, non-XB mechanisms are not merely minor extensions of classical models of muscle contraction (Hill, 1938; Huxley, 1957), but represent important contributions to muscle function during repeated cyclic loading. As such, the proposed ‘sarcomeric memory’ may have relevance across diverse muscle scales, muscle types and locomotor strategies, providing a mechanistic framework that can now be tested in a variety of settings. Specifically, varying the ICI, activation level, stretch velocity and number of SSCs may help identify the conditions that promote or limit performance-enhancing carryover effects and determine whether, and under what conditions, these effects saturate.

### Limitations

The stretch velocity used in the present study (1% *v*_max_) is considerably lower than velocities typically encountered during daily activities or sports-related movements. However, reproducing physiologically representative movement velocities was not the primary aim of the present experiments. Rather, the use of 1% *v*_max_ was selected to provide experimental conditions comparable to our previous study (Elst et al., 2025) and to optimise the experimental setup for isolating and investigating carryover effects across consecutive SSCs. Although slow and controlled SSCs with intermittent isometric phases can occur during resistance training, the extent to which the observed carryover effects translate to faster and more dynamic SSCs remains to be investigated.

A further limitation is related to the precise cellular basis underlying the increase in SRS. A stretch-induced reduction in lattice spacing could transiently favour actin–myosin interaction; however, fibres were returned to their initial length before the next SSC, meaning that a purely transient change in lattice spacing alone cannot readily explain a stiffness increase that persists across cycles. Whether such transient changes additionally trigger longer-lasting structural or mechanosensory adaptations remains to be determined. Similarly, our experimental design cannot distinguish between the specific contributions of weakly bound XBs, low-force strongly bound XBs and additional XB recruitment via mechanosensing. The interpretations offered above should therefore be read with this uncertainty in mind.

### Conclusion

Repeated slow SSCs separated by brief deactivation phases progressively increased peak force and mechanical work. These enhancements persisted under XB inhibition, highlighting a substantial contribution from non-XB structures, with titin being a likely candidate. In the control condition, increased short-range stiffness (SRS) reflected XB-based carryover effects, whereas under blebbistatin, force and work rose without changes in SRS. Together, these results indicate that the stretch phase of SSCs creates a ‘sarcomeric memory’ effect, leading to persistent changes in both XB and non-XB elements that enhance work during stretching across the following cycles. Future work should examine how activation/deactivation timing and contraction velocity shape these cycle-to-cycle carryover effects and determine whether they eventually reach saturation with repeated SSCs.

## Supplementary Material

10.1242/jexbio.252866_sup1 Supplementary information
