# Supplementary material for: Repeated stretch–shortening cycles enhance force and work despite brief deactivation between cycles
Source: J Exp Biol. 2026 Oct 9;229(19):jeb252866. doi: 10.1242/jeb.252866 (PMC13723387; doi:10.1242/jeb.252866)
Supplement: Supplementary file 1 [file jexbio-229-252866-s1.pdf]

Supplementary Materials and Methods

Animal handling, tissue preparation and solutions

Extensor digitorum longus (EDL) muscles were obtained from three male Wistar rats (8–10 months old, 300–350 g, cage-sedentary, 12 h:12 h light: dark cycle, housing-temperature: 22 °C). The animals were euthanised via CO_2 overdose. All procedures complied with the ARRIVE guidelines and were approved in accordance with German animal protection law (Tierschutzgesetz, §4 (3); Permit No. T 236/24). Muscle preparation, storage and activation techniques for permeabilized single muscle fibres followed Tomalka et al. (2017, 2020).

Fibre bundles (approx. 50-100 fibres) were cut from the EDL muscle, permeabilized in skinning solution (see **Table S1**) containing 2% (vol/vol) Triton X-100 for 30 minutes, then rinsed for 30 minutes in relaxing solution and placed in a storage solution (1:1 glycerol–skinning solution mixture) at −20 °C for up to 6–8 weeks. On the day of the experiments, single fibres were isolated from the skinned fibre bundles under a stereomicroscope (Leica, Model A60, Germany) using fine forceps. Fibres (n = 21) were then trimmed to a length of 1.04 ± 0.13 mm and clamped at both ends using aluminium foil T-shaped clips. To ensure complete removal of internal membranes without affecting the contractile apparatus, the fibre-clip preparations were additionally placed in a skinning solution (relaxing solution supplemented with 1% (vol/vol) Triton X-100 for 1–2 min at 4°C (Fryer et al., 1995; Linari et al., 2007)).

Journal of Experimental Biology • Supplementary information

Experimental setup

Single muscle fibres were transferred from the skinning solution into the experimental chamber of a permeabilized fibre test system (Aurora Scientific, Model 1400A, Canada) filled with relaxing solution. The fibre-clip assembly was mounted on hooks attached to a force transducer (Aurora Scientific, Model 403A; resolution: 0.1 µN) and a high-speed length controller (Aurora Scientific, Model 322C-I; resolution: 0.5 µm). To improve stability and prevent any slippage during contractions, the clips were secured to the hooks using a thin layer of fingernail polish. The apparatus was positioned on the x–y stage of an inverted microscope (Eclipse Ti, Nikon, Japan) to allow continuous visual monitoring. Sarcomere length was measured in the central region of the fibre in the passive state and adjusted to 2.5 ± 0.05 µm. The corresponding fibre length was defined as the optimal length (L_{opt}) at which maximal steady-state isometric force (F_0) is produced (Stephenson & Williams, 1982; Tomalka et al., 2017). Fibre width (*w*) and height (*h*) were recorded at 0.1-mm intervals along the fibre using a 10× ELWD dry objective (NA 0.60, Nikon, Japan) and a 10× eyepiece. The cross-sectional area was estimated assuming an elliptical geometry ($\pi \times h \times w/4$) and averaged 0.0054 ± 0.0010 mm². All experiments were conducted at a bath temperature of 12 ± 0.1 °C, as this temperature provides optimal fibre stability during active stretching and prolonged activations (Ranatunga, 1982, 1984). Fibre activation was induced by Ca^{2+} diffusion (pCa 4.5) in the presence of ATP. Cross-bridge (XB) inhibition was achieved with Blebbistatin. Blebbistatin is a photosensitive myosin II inhibitor that blocks the force-generating transition of the actomyosin complex, thereby preventing the formation of strongly bound XBs while still permitting weakly bound states that contribute to stiffness rather than force (Bond et al., 2013). Importantly, titin mobility is not affected by Blebbistatin (Shalabi et al., 2017).

Journal of Experimental Biology • Supplementary information

Table S1. Compositions of the solutions. Concentrations are given in mM, with the exception of glycerol (v/v) and creatine phosphokinase (**CK;** U ml^{-1}). **TES**: N-tris[hydroxymethyl]methyl-2-aminoethanesulphonic acid; **ATP**: adenosine 50-triphosphate disodium salt hydrate; **EGTA**: ethylene glycol-bis(2-aminoethylether)-N,N,N0,N0-tetraacetic acid; **CP**: N-[Imino(phosphonoamino) methyl]-N-methylglycine; **GLH**: glutathione; **HDTA**: 1,6 diaminohexane-N,N,N0,N0-tetraacetic acid; **KP**: potassium propionate; **IMID**: imidazole; **PMSF:** phenylmethanesulfonyl fluoride. To all solutions, cysteine and cysteine/serine protease inhibitors (10 mM **E-64**: trans-epoxysuccinyl-L-leucylamido-(4-guanidino)butane and 20 µg ml^{-1} leupeptin, respectively) were added to preserve the lattice proteins and maintain the homogeneity of the sarcomeres (Linari et al., 2007; Tomalka et al., 2017). The pH value, adjusted with potassium hydroxide (**KOH**), was 7.1 at 12 °C. The ionic strength was 190 mM. CK was obtained from Roche (Mannheim, Germany), and Blebbistatin was obtained from Enzo Life Sciences Inc. (NY, United States). The other chemicals used in our study were obtained from Sigma (St. Louis, MO, USA). The solutions used during the experiments were identical to those described in (Linari et al., 2007).

	**TES**	**$MgCl_2$**	**Na_2ATP**	**EGTA**	**Na_2CP**	**GLH**	**HDTA**	**CaEGTA**	**CK (U ml^{-1})**
**Relax**	100	7.70	5.44	25	19.11	10	-	-	400-500
**Pre-activation**	100	6.93	5.45	0.1	19.49	10	24.9	-	400-500
**Activation**	100	6.76	5.46	-	19.49	10	-	25	400-500
	**KP**	**$MgCl_2$**	**Na_2ATP**	**EGTA**	**IMID**	**GLH**	**PMSF**	**Glycerol**	**CK (U ml^{-1})**
**Skinning**	170	2.5	2.5	5	10	-	0.2	-	-
**Storage**	170	2.5	2.5	5	10	10	0.2	50%	-

Journal of Experimental Biology • Supplementary information

References

Bond, L. M., Tumbarello, D. A., Kendrick-Jones, J., & Buss, F. (2013). Small-Molecule Inhibitors of Myosin Proteins. *Future Medicinal Chemistry*, *5*(1), 41–52. https://doi.org/10.4155/fmc.12.185

Fryer, M. W., Owen, V. J., Lamb, G. D., & Stephenson, D. G. (1995). Effects of creatine phosphate and P(i) on Ca2+ movements and tension development in rat skinned skeletal muscle fibres. *The Journal of Physiology*, *482*(1), 123–140. https://doi.org/10.1113/jphysiol.1995.sp020504

Linari, M., Caremani, M., Piperio, C., Brandt, P., & Lombardi, V. (2007). Stiffness and Fraction of Myosin Motors Responsible for Active Force in Permeabilized Muscle Fibers from Rabbit Psoas. *Biophysical Journal*, *92*(7), 2476–2490. https://doi.org/10.1529/biophysj.106.099549

Ranatunga, K. W. (1982). Temperature-dependence of shortening velocity and rate of isometric tension development in rat skeletal muscle. *The Journal of Physiology*, *329*(1), 465–483. https://doi.org/10.1113/jphysiol.1982.sp014314

Ranatunga, K. W. (1984). The force-velocity relation of rat fast- and slow-twitch muscles examined at different temperatures. *The Journal of Physiology*, *351*(1), 517–529. https://doi.org/10.1113/jphysiol.1984.sp015260

Shalabi, N., Cornachione, A., de Souza Leite, F., Vengallatore, S., & Rassier, D. E. (2017). Residual force enhancement is regulated by titin in skeletal and cardiac myofibrils. *The Journal of Physiology*, *595*(6), 2085–2098. https://doi.org/10.1113/JP272983

Stephenson, D. G., & Williams, D. A. (1982). Effects of sarcomere length on the force—pCa relation in fast- and slow-twitch skinned muscle fibres from the rat. *The Journal of Physiology*, *333*(1), 637–653. https://doi.org/10.1113/jphysiol.1982.sp014473

Tomalka, A., Rode, C., Schumacher, J., & Siebert, T. (2017). The active force–length relationship is invisible during extensive eccentric contractions in skinned skeletal muscle fibres. *Proceedings of the Royal Society B: Biological Sciences*, *284*(1854), 20162497. https://doi.org/10.1098/rspb.2016.2497

Tomalka, A., Weidner, S., Hahn, D., Seiberl, W., & Siebert, T. (2020). Cross-Bridges and Sarcomeric Non-cross-bridge Structures Contribute to Increased Work in Stretch-Shortening Cycles. *Frontiers in Physiology*, *11*. https://doi.org/10.3389/fphys.2020.00921
